# Minimum acceptable diet intake and its associated factors among children age at 6–23 months in sub-Saharan Africa: a multilevel analysis of the sub-Saharan Africa demographic and health survey

**DOI:** 10.1186/s12889-022-12966-8

**Published:** 2022-04-07

**Authors:** Daniel Gashaneh Belay, Asefa Adimasu Taddese, Kasahun Alemu Gelaye

**Affiliations:** 1 Department of Human Anatomy, School of Medicine, College of Medicine and Health Sciences, University of Gondar, Gondar, Ethiopia; 2 Department of Epidemiology and Biostatistics, Institute of Public Health, College of Medicine and Health Sciences, University of Gondar, Gondar, Ethiopia

**Keywords:** Minimum acceptable diet, Socioeconomic inequalities, Sub-Saharan African

## Abstract

**Background:**

Only one in five children aged below 24 months in the low-income countries feed the minimum recommended diet, and significantly varied across socio-economic classes. Though sub-saharan Africa (SSA) shares the huge burden of children under nutrition, as to our search of literature there is limited evidence on the pooled magnitude and factors associated with minimum acceptable diet (MAD) intake among children aged 6 to 23 months in the region. This study aimed to assess the pooled magnitude and associated factors of MAD intake among children aged 6–23 months in SSA using recent 2010–2020 DHS data.

**Methods:**

Demographic and Health Survey datasets of SSA countries were used for this study with a total of 78,542 weighted samples. The data were cleaned using MS excel and extracted and analyzed using STATA V.16 software. A multilevel binary logistic regression model was fitted. The adjusted odds ratio (AOR) with *P*-value < 0.05 was taken to declare statistical significance.

**Results:**

The pooled magnitude of MAD intake among children aged 6–23 months in SSA was 9.89% [95%CI: 8.57, 11.21%] ranging from 3.10% in Guinea to 20.40% in Kenya. Individual level factors such as; secondary &above women educational status [AOR = 1.41; 95%CI; 1.29, 1.53], having employed women [AOR = 1.25;95%CI;1.17,1.33], having media exposure [AOR = 1.55;95%CI;1.45,1.66], richest household wealth [AOR = 1.93; 95%CI; 1.73, 2.15], plural birth [AOR = 0.68;95%CI; 0.56, 0.82] and breastfed child [AOR = 2.04; 95%CI; 1.89, 2.21], whereas, community level factor such as rural residence [AOR = 0.74; 95%CI; 0.69, 0.79] and living in upper middle income country [AOR = 1.62; [95%CI; 1.41,1.87] were significantly associated with MAD intake.

**Conclusion and recommendations:**

Minimum acceptable diet intake in SSA is relatively low. Variables such as; secondary &above maternal education, having employed mother, exposure to media, richest wealth, breast feeding child, and upper middle income country have a significant positive association, whereas having plural birth and living in rural residence have a significant negative association with MAD intake. These findings highlight that policymakers and other stakeholders had better give prior attention to empowering women, enhance household wealth status and media exposure to increase the MAD intake in the region.

**Supplementary Information:**

The online version contains supplementary material available at 10.1186/s12889-022-12966-8.

## Introduction

### Background

Nutrition has profound effects on health throughout the human life course and is inextricably linked with cognitive and social development, especially in early childhood [1, 2]. In settings with insufficient material and social resources, children cannot able to access their full recommended diet based on their age status [1].

Undernutrition (stunting and wasting) and hidden hunger (deficiencies in essential vitamins and minerals) are the most common among the three strands of children’s malnutrition [3, 4]. To overcome these problems, in children who are 6 months of age and onwards, complementary feeding needs to be started [2]. Complementary feeding is the process of introduction of other foods and liquids along with breast milk for 6–23 months aged children when breast milk can no longer sufficiently meet the required nutritional for infants [5]. Minimum acceptable diet (MAD) is a combination of both minimum meal frequency and minimum dietary diversity and one of the eight indicators of infant and young child feeding (IYCF) practices launched by World Health Organization (WHO) [6, 7]. Minimum dietary diversity is a proxy for adequate micronutrient density of foods and defined as children who received four or more from the following seven food groups [7, 8]. These are Grains, roots and tubers, legumes and nuts, dairy products (milk, yogurt, and cheese), flesh foods (meat, fish, poultry, and liver/organ meats), eggs, vitamin-A rich fruits and vegetables and other fruits and vegetables [2, 9, 10]. Whereas, minimum meal frequency intake is a proxy for meeting energy requirements and defined as daily consumption of ≥2 times for children aged 6–8 months, ≥3 times for age of 9–23 months, and ≥ 4 times for those who were not breastfed [2, 6, 9, 11].

Global estimates for feeding of children aged 6–23 months indicate substantial room for improvement [4, 5]*.* In many countries, less than one-quarter of children are reported not getting the nutrition they need to grow well, particularly in the crucial first 1000 days [3, 12]. Child under nutrition is a major public health problem in many resource-poor communities in the world [13]. Only one in five children aged 6 to 23 months from the poorest households and rural areas is fed the minimum recommended diverse diet [3]. The magnitude of minimum acceptable diet (MAD) usage among children aged 6–23 months in India, ranges from 4 to 9% whereas, in the Philippines, only 6.7% fulfill minimum acceptable diet standards [14, 15]. Studies conducted in Bangladesh and Indonesia showed that 20 and 40% of children get MAD respectively [16, 17].

Suboptimal IYCF practices remain serious public health concerns in children [13]. The first 1000 days (time from conception up to 2 years of age) of the child’s life provide a critical window of opportunity to ensure survival, growth, and development through optimum infant and young child feeding (IYCF) practices [5]. Inappropriate IYCF practices during this period result in significant threats to child health by impaired cognitive development, compromised educational achievement, and low economic productivity which become difficult to reverse later in life [5, 10, 18]. More than two-thirds of malnutrition related child deaths are associated with inappropriate feeding practices during the first 2 years of life [19]. Undernutrition is linked to just under half of all deaths of children aged under 5 in each year [3, 5]. Nearly one-third of child deaths could be prevented by optimal complementary feeding practices [20]. Research has shown that in sub-Saharan Africa children lost up to 2.5 years of schooling if there was a famine while they were in utero and during their childhood [3].

From individual level variables, socio demographic factors such as; maternal education [13, 21, 22], marital status [13, 15], occupation of mothers [13, 23, 24], and wealth status of the households [21, 22], child related characteristic such as; child age [20, 22, 25], child sex [15], birth order [15, 22, 26], and plurality of birth [27], health service utilization factors such as; having institutional delivery [15, 20, 24, 26, 28], having growth monitoring follow up [5, 20], antenatal care visit (ANC) [15], and postnatal care visits (PNC) [5, 10, 24], behavioral factors: such as household media exposure [15, 21, 23] have a significant association with MAD usage. Whereas community level variables such as; living in urban areas [13, 21, 23, 29], and regional variation [15, 25, 29] have a significant association with MAD usage among children aged 6–23 months.

**Fig. 1:**
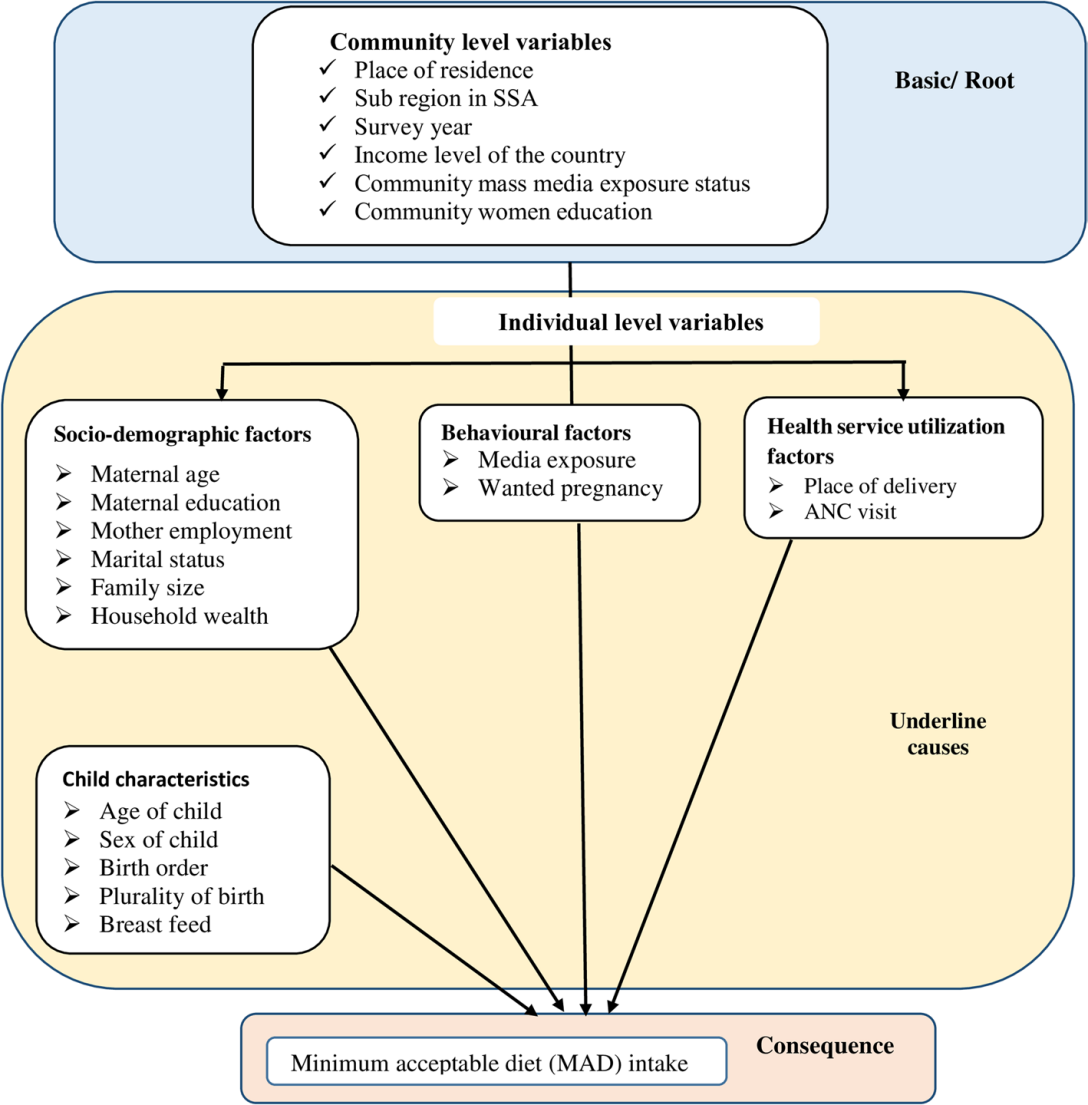
Conceptual framework of factors associated with Minimum acceptable diet (MAD)

The conceptual framework in Fig. 1 shows individual and community level factors which directly or indirectly associated with MAD intake among children aged 6–23 months. They are basic or root causes and underline causes based on nutritional frameworks [3, 5, 15, 24, 25] [Fig. 1].

Most of the previous studies conducted on the prevalence and associated factors of MAD usage were conducted in a single pocket setting [5, 19, 20, 30], which is unable to determine the variation of MAD between clusters. But this study conducted on 33 Sub-Saharan Africa DHS data which have 2 stages (two levels) and cover a wider number of enumeration areas which made it easy to use multi-level analysis to estimating random effect sizes. The data were also be weighted using a weighting variable (v005), and primary sampling unit/cluster (v001), then the weighted data have been used for all analyses to get a reliable estimate and standard error to draw a valid conclusion.

There have been studies reporting the burden and determining factors of childhood MAD usage in different countries of Sub-Saharan Africa. But these studies were varied in the regional variation of local food items and food cultures which makes difficult to make regional comparisons. Moreover factors associated with minimum acceptable diet intake are complex and interwoven each other which operating simultaneously. Therefore, this study used the most recent standard DHS dataset which was collected in a similar design and standardized parameters which makes easy to multilevel analysis the magnitude and associated factors of MAD intake.

Poor practices of minimum acceptable diet have a determinate impact for almost all health and growth in children less than 2 years of age which has been recognized as the ‘critical window period”. Therefore, this pooled magnitude of MAD will be the crucial point for policy makers to know child nutrition status in the sub-Saharan African region and to draft child nutrition policy and take actions based on the results.

Therefore this study has been attempt to answer the following major research questions. What was the pooled magnitude of minimum acceptable diet usage among children aged 6–23 months in SSA and its regional distribution?, What are the common factors associated with minimum acceptable diet intake among children 6–23 months aged in SSA countries?

## Methods

### Study design, setting, and period

The data source for this study was the recent standard DHS data of Sub-Saharan African countries conducted within 10 years (2010–2020), which was a crossectional study conducted every 5 year interval to generate updated health and health-related indicators. In this study, 10 years of DHS data (starting from 2010) were taken to analyze for two reasons. The first is because the currently used IYCF (infant and young children feeding) assessment guidelines was issued by WHO in 2010 even if the first draft was released by 2007 [6, 7]. Whereas, the second reason is to get a representative sample of recent standard DHS data from each region of SSA. The standard DHS data set was used for each country to get all parameters and a large sample size which can be representative of the source of population and have particular advantage for cross-country analysis [9].

**Fig. 2:**
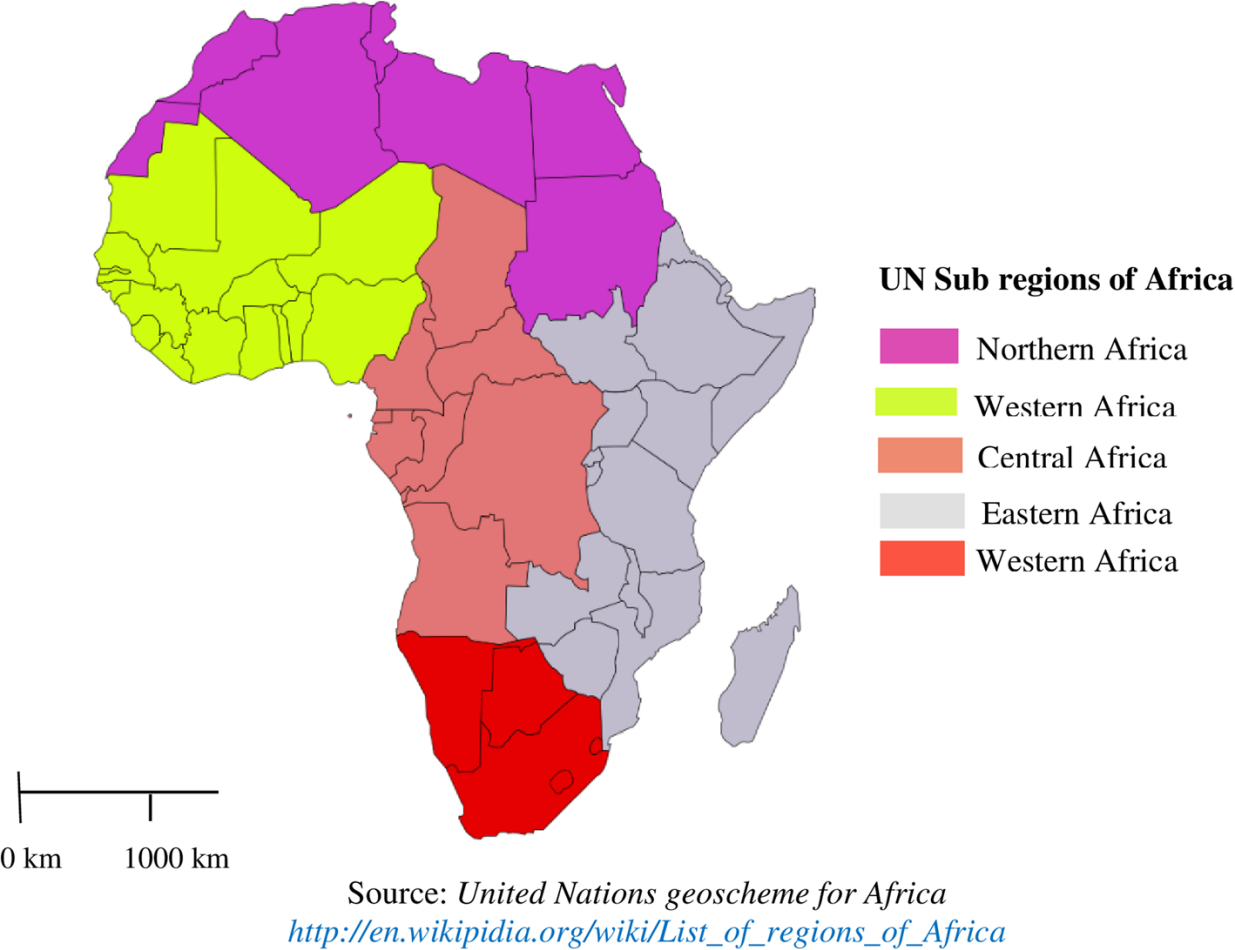
The map of the study area (Sub-Saharan African countries) adapted from united nation geoscheme for Africa

The Sub-Sahara is the area in the continent of Africa that lies south of the Sahara and consists of four vast and distinct regions. A total of 33 Sub-Saharan Africa countries were represented for this study in the four regions namely Eastern Africa (Burundi, Comoros, Ethiopia, Kenya, Malawi, Mozambique, Rwanda, Tanzania, Uganda, Zambia, Zimbabwe), Central Africa (Angola, Cameroon, Chad, the Democratic Republic of the Congo, Republic of the Congo, Gabon), Western Africa (Benin, Burkina Faso, Ivory Coast, Gambia, Ghana, Guinea, Liberia, Mali, Niger, Nigeria, Senegal, Sierra Leone, Togo), and Southern Africa (Lesotho, Namibia, South Africa) [31] (Fig. 2). Together, they constitute an area of 9.4 million square miles and a total population of 1.1 billion inhabitants [32]. The datasets are publicly available from the DHS website www.dhsprogram.com [31]. DHS collects data that are comparable across countries. The surveys are nationally representative of each country and population-based with large sample sizes. All surveys use a multi-stage cluster sampling method [9] [Fig. 2].

### Populations

#### Source population

The source population were all children aged 6–23 months preceding 5 years of the survey period across 33 Sub-Saharan Africa countries.

#### Study population

The study population were children aged 6–23 months preceding 5 years the survey period in the selected Enumeration Areas (EAs) which is the primary sampling units of the survey clusters. The mother or the caregiver was interviewed for the survey in each country and mothers who had more than one child within the 2 years preceding the survey were asked questions about the most recent child [8].

#### Inclusion criteria

All children aged 6–23 months preceding 5 years of the survey in the selected EAs in each SSA country were included in this study.

#### Exclusion criteria

Five countries that did not have a survey report after the 2010/2011 survey year were excluded due to the recent updates. These countries are Central Africa Republic (DHS report 1994/95), Eswatini (DHS report 2006/07), Sao Tome Principe (DHS report 2008/09), Madagascar (DHS report 2008/09), and Sudan (DHS report 1989–90). As well as three Sub-Saharan Countries (Botswana, Mauritania, and Eritrea) were excluded due to the dataset not being publicly available.

Moreover from the included countries data set, children in the age category of 6–23 months which is not assessed to MAD based on the DHS guideline, and having missing value of the outcome variable were excluded.

### Sample size determination and sampling method

#### Sample size determination

In general, the most recent census frame was used in all of the surveys conducted in the selected countries. Typically, DHS samples are stratified by administrative geographic region and by urban/rural areas within each region. DHS sample designs are usually two-stage probability samples drawn from an existing sample frame. In the first stage of sampling, Enumeration Areas (EAs) were selected with probability proportional to size within each stratum. In selected EAs, a fixed number of households is selected by systematic sampling method in the second stage of sampling. Following the listing of the households, a fixed number of households is selected by equal probability systematic sampling in the selected cluster [9]. The detailed sampling procedure was available in each DHS reports from the Measure DHS website (www.dhsprogram.com) [9].

The children’s records or kid’s records (KR) DHS datasets were used. Weighted values were used to restore the representativeness of the sample data. Since the overall probability of selection of each household is not a constant, before using the DHS dataset it must be weighted. DHS guideline set four sampling weighting methods and from that, the individual weight for women (v005) which is the household weight (hv005) multiplied by the inverse of the individual response rate for women in the stratum was used. Individual sample weights are generated by dividing (v005) by 1000,000 before use to approximate the number of cases [33].

Finally, a total weighted sample of 78, 542 children in the age category of 6–23 months from all 33 countries were included in this study [Table 1].

**Table 1:** Sample size determination of MAD intake and factor associated with it among children aged 6–23 months in each Sub-Saharan Africa: based on 2010–2020 DHS

Sub-Saharan Africa Countries with Recent DHS report from 2010/11 to 2019/20				
			Sample size	
Regions	Country	DHS year	Un weighted	Weighted
East Africa countries	Burundi	2016/17	4016	4145
	Comoros	2012	727	728
	Ethiopia	2016	2850	3032
	Kenya	2014	2822	2610
	Malawi	2015/16	4642	4664
	Mozambique	2011	3158	3312
	Rwanda	2014/15	1133	1159
	Tanzania	2015/16	3159	3105
	Uganda	2016	4391	4327
	Zambia	2018	2851	2780
	Zimbabwe	2015	1545	1599
Central Africa countries	Angola	2015/16	4020	3706
	Cameroon	2018	2673	2771
	Chad	2014/15	2791	2878
	DR Congo	2013/14	2572	2495
	Congo	2011/12	1504	1339
	Gabon	2012	1112	875
West Africa countries	Benin	2017/18	3965	3968
	Burkina Faso	2010	2080	2099
	Ivory Coast	2011/12	1095	1090
	Gambia	2013	1160	1134
	Ghana	2014	879	864
	Guinea	2018	1917	1867
	Liberia	2019/20	1538	1359
	Mali	2018	2751	2901
	Niger	2012	1523	1588
	Nigeria	2018	9211	9292
	Senegal	2019	1349	1262
	Sierra Leone	2019	2685	2669
	Togo	2013/14	1063	1037
Southern Africa countries	Lesotho	2014	468	464
	Namibia	2013	644	596
	South Africa	2016	843	827
	**Total sample size**	**79,147**	**78, 542**	

#### Sampling procedures

**Fig. 3:**
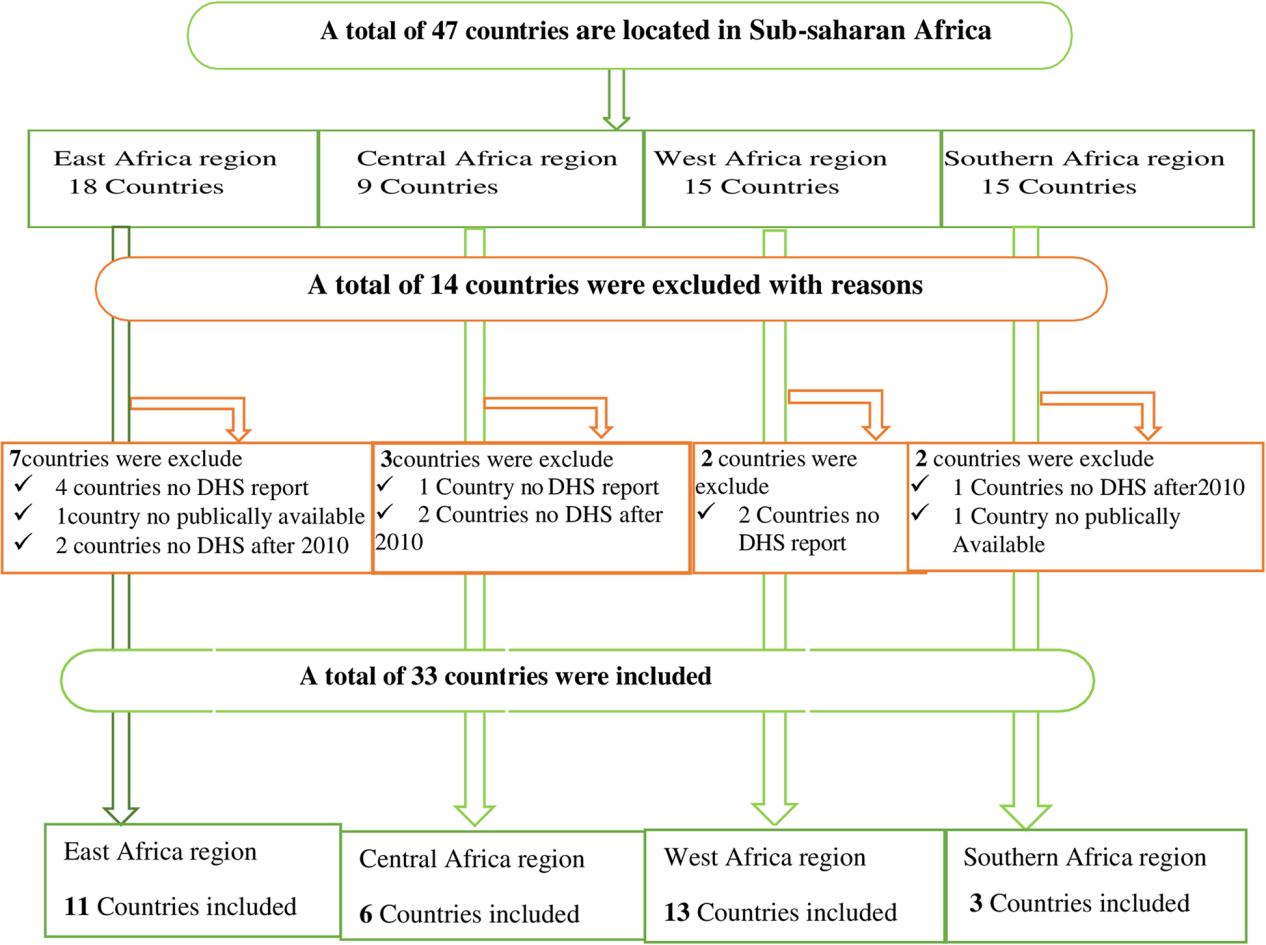
Sampling procedures to select the eligible countries for the study

A total of 47 countries are located in Sub-Saharan Africa. Of these countries, only 41 countries had demographic and health survey report. Then, after excluding countries that had no DHS report after 2010 and countries where the DHS dataset was not publicly available, a total of 33 countries were included in this study. From the total 81,123 eligible households, 79,147 had complete data for MAD intake with response rate of 97.6%. The details of the sampling procedure summarized in Fig. 3 below [Fig. 3].

#### Study variables

##### Dependent variables

The outcome variable of this study is taking minimum acceptable diet (MAD) of children 6–23 months aged which is combined from children who had minimum meal frequency and minimum dietary diversity in both breastfeeding and non-breastfeeding children. During the survey, their mother was asked questions about the types and frequency of food the child had consumed during the day or night before the interview [9].

If a child is taken four out of seven food groups fed during the day or night preceding the survey from the following food items are considered as getting minimum dietary diversity. These are Grains, roots and tubers, Legumes and nuts, Dairy products (milk, yogurt, and cheese), Flesh foods (meat, fish, poultry and liver/organ meats), Eggs, Vitamin A rich fruits and vegetables, and other fruits and vegetables. Whereas minimum meal frequency is the provision of two or more feedings of solid, semi-solid, or soft food for 6–8 months, three or more feedings for 9–23 months breastfeed, and four times for non-breastfed children. The data of the above variables were collected similarly across all SSA countries [7, 9]. Since minimum meal frequency has a different cut off value for different age groups and breastfed and non-breast feed children, so as the overall meal frequency computed after computing for each group.

##### Independent variables

Individual and community-level independent variables have been studied. The individual-level factors include socio-demographic characteristics such as; the age of the mother, mother employment, marital status, family size, maternal education, and household wealth status were included. Child-related factors such as the age of the child, sex of the child, birth order, the plurality of birth, and breastfeeding status of the child are all taken into account.

Behavioral characteristics like media exposure were studied. Media exposure status was created from the frequency of watching TV, listening to the radio, and reading a newspaper or magazine. If a woman has at least one yes she has considered media exposed. Health service utilization-related factors such as place of delivery, ANC visit also considered.

The community-level factors include; the place of residence, region in sub-Saharan Africa, survey year, country income level, community-level media exposure, and community-level women education were considered.

Community-level media exposure was assessed by the proportion of women who had at least been exposed to one media, television, radio, or newspaper. It was coded as “0” for low(communities in which < 50% women had media exposure at least for one media), “1” for high community-level media exposure (communities in which ≥50% women had at least for one media [34, 35]. Community-level women’s education was also assessed by the proportion of women who had at least primary education. It was coded as “0” for low(communities in which < 50% women had at least primary education), “1” for high community-level women education (communities in which ≥50% women had at least primary education (at cluster level) [34, 35].

The countries income status was categorized as low income, lower middle income, and upper-middle-income country based on the World Bank List of Economies classification since 2019 [36]. World Bank calculated country income based on Gross National Income (GNI) per capita, which categorized as low income $1025 or less; lower middle income, $1026-3995, upper middle income $3996-12,375,and high income $12,375 or more [36]. Survey year mean the recent standard DHS data collection period of each countries from 2010 to 2020.

#### Data processing and analysis

This study was performed based on the DHS data obtained from the official DHS measure website www.measuredhs.com after permission has been obtained via an online request by specifying my objectives. Data from the DHS dataset were downloaded standard DHS data in STATA format then cleaned, integrate, transformed, and append to produce favorable variables for the analysis. Microsoft excel and STATA 16 software were used to generate both descriptive and analytic statistics of the appended 33 countries data to describe variables in the study using statistical measurements. The pooled estimate of MAD intake among children in Sub- Saharan Africa and Sub-regions was estimated using a *metan* STATA command.

#### Model building for multi-level analysis

The DHS data has hierarchical nature, and children aged 6 to 23 months and mothers were nested within a cluster. This might violate the standard logistic regression model assumptions such as the independence and equal variance assumptions. Therefore, a multilevel binary logistic regression model was fitted. Four models were fitted for multi-level analysis. The first was the null model (Model 1) containing no exposure variables which were used to check the variability of MAD across the cluster. The second (Model 2) and the third (Model 3) multilevel models contain individual-level variables and community-level variables respectively. In the fourth model (Model 4), both individual and community level variables were fitted simultaneously with the prevalence of MAD. Model comparisons were done using the deviance test and log likelihood test and the model with the highest log likelihood ratio and the lowest deviance was selected as the best-fitted model. The variance inflation factor (VIF) was used to detect multicollinearity, and all variables had VIF values less than 10 and the mean VIF value of the final model was 1.54.

#### Parameter estimation method

**The fixed effects (a measure of association)** were being used to estimate the association between the likelihood of prevalence of MAD intake and explanatory variables at both individual and community levels. Associations between dependent and independent variables were assessed and its strength has been presented using Adjusted Odds Ratio (AOR) and 95% confidence intervals with a *p*-value of < 0.05.

$$Log\ \left(\frac{\pi ij}{1-\pi ij}\right)=\beta o+\beta 1 xij+\beta 2 xij+\dots uj+ eij$$

Where, *πij*: the probability of MAD use, 1 − *πij*: the probability of no MAD use, b1xij are individual and community level variables for the ith individual in group j, respectively. The ß’s are fixed coefficients indicating a unit increase in X can cause a ß unit increase in probability feeding MAD. While the ß0 is intercept that is the effect on feeding MAD when the effect of all explanatory variables are absent. The uj shows the random effect (effect of the community on the mother’s decision to provide MAD) for the j^th^ community. The clustered data nature and the within and between community variations were taken in to account assuming each community has a different intercept (β0) and fixed coefficient (β) [35, 37, 38].

**Random effects (a measure of variation)** were estimated by the median odds ratio (MOR), Intra Class Correlation Coefficient (ICC), and Proportional Change in Variance (PCV).

The MOR is defined as the median value of the odds ratio between the area at the highest risk and the area at the lowest risk when randomly picking out two clusters.

MOR = exp.[√(2 × VA) × 0.6745], or $\mathrm{MOR}={e^{0.95}}^{\sqrt{VA}}$ where; VA is the area level variance [35, 37, 39].

The PCV reveals the variation in the prevalence of MAD intake among children 6–23 months explained by factors. The PCV is calculated as; $PCV=\frac{Vnull- VA}{V\ null}\ast 100\%$ where; Vnull = variance of the initial model, and VA = variance of the model with more terms.

The ICC which reveals the variation of MAD between clusters is calculated as; $\kern0.5em ICC=\frac{VA}{VA+3.29}\ast 100\%$, where; VA = area/cluster level variance [35, 37, 39].

#### Data quality control

The DHS data are comparable across countries. The missing values were clearly defined by the DHS guide line. If there were missing values and “don’t know” in breastfeeding, assumed as not breast feeding, but if there were in specific foods, excluded from further analysis [9]. If the missing value in explanatory variables were greater than 5%, since the DHS survey is a crossectional study dropping the variables from the further analysis were done. The data extractions were performed by public health experts who have experience with DHS data to ensure the quality of data. The data were cleaned and extracted from the kid’s record (KR) file in standard DHS. Before appending all 33 countries’ data, each country’s data consistency has been cheeked.

All variables measured in DHS were measured consistently with similar interview questions. But there were some variables coded by different codes in some SSA countries DHS, like child age, and giving cereal foods. So during data management, they recoded uniformly with others countries to compile easily. The data have been checked for consistency and completeness daily by the supervisor and principal investigator. The magnitude of MAD usage among children in each country was compared with the respective DHS reports.

#### Ethical clearance

After the consent paper was submitted to DHS Program/ICF International Inc., a letter of permission was waived from the International Review Board of Demographic and Health Surveys (DHS) program data archivists to download the dataset for this study**.** The dataset was not shared or passed on to other bodies and has maintained its confidentiality.

## Results

### Socio demographic characteristics of mothers or care givers and the children

A total weighted sample of 78,542 children of age 6–23 were included in this study. Three fourths (75.52%) of mothers of children were in the age group of 20–35 years, with a median age of 27 (IQR: 16) years. More than three-fifths of women (61.7%) had formal education. Most (70%) of the mothers were not working. Moreover, 66.46% of children were delivered at the health facility.

Almost equal proportions of male (50.49%) and female (49.51%) children were studied. Nearly two-thirds (65.42%) of the children were found in the age group from 12 to 23 months with a median age of 14 (IQR: 8) months. Almost all (96.63%) of child birth were singleton. Four-fifths (79.49%) of the children were breastfeeding.

From the community level variables, most (70%) of the respondents were rural inhabitants. Sixty-four percent of the sub-Saharan African countries included in the study were lower-income countries (Table 2).

**Table 2:** Socio-demographic characteristics of the smothers/care givers and the children in a study of MAD intake and associated factors among children 6–23 months aged in Sub-Saharan Africa: based on 2010–2020 DHS

Variables	Categories	Frequency (n)		Weighted Percentage (%)
		Unweighted	Weighted	
Socio-demographic characteristics and health service utilization of the mothers				
Age of women (years)	15–19	7500	7305	9.30
	20–35	59,774	59,541	75.81
	36–49	11,873	11,696	14.89
Sex of household head	Male	60,808	60,929	79.71
	Female	16,259	15,513	20.29
Educational attainment of women	No education	30,314	29,673	37.78
	Primery education	27,104	26,888	34.23
	Secondary & above	21,729	21,981	27.99
Occupation of women	Worked	23,008	22,214	29.44
	Not working	53,010	53,239	70.56
Marital status of mother	Married	55,160	55,286	70.39
	Not married	23,987	23,255	29.61
House hold family size	1–4	21,823	22,124	28.17
	5–10	47,473	46,784	59.57
	> 11	9851	9633	12.26
Media exposure	No	30,381	29,187	37.21
	Yes	48,670	49,261	62.79
Pregnancy wontedness	Wanted	56,314	55,767	71.02
	Unwanted	22,820	22,765	28.98
Place of delivery	Home delivery	26,544	52,586	33.04
	Health facilities	52,598	25,956	66.96
ANC visits	No ANC	12,715	12,304	16.07
	At least one ANC	66,432	66,238	83.93
Child related characteristics				
Sex of child	Male	40,056	39,657	50.49
	Female	39,091	38,885	49.51
Age of child	6–8 months	14,301	14,097	17.95
	9–11 months	13,035	13,066	16.64
	12–23 months	51,811	51,378	65.42
Birth order	< three	44,745	44,659	56.86
	> three	34,402	33,882	43.14
Plurality	Single	76,478	75,940	96.69
	Multiple	2669	2602	3.31
Breast feeding status	Not breastfed	16,232	16,039	20.51
	breastfed	62,915	62,503	79.49
Community level variables				
Residence	Urban	23,782	24,533	31.24
	Rural	55,365	54,009	68.76
Region in SSA	Central Africa	14,682	14,064	17.91
	East Africa	31,294	31,462	40.06
	West Africa	31,216	31,130	39.64
	South Africa	1955	1886	2.40
Country income level	Lower	51,015	51,329	64.46
	Lower middle	21,503	21,209	27.17
	Upper middle	6629	6003	8.38
Survey year	2010–2012	12,558	12,292	15.87
	2013–2015	26,898	26,310	33.98
	2015–2019	39,691	39,939	50.15
Community media exposure	Low	39,529	38,619	49.17
	High	39,618	39,923	50.83
Community women education	Low	39,567	38,640	49.2
	High	39,580	39,901	50.8

### Pooled magnitude of minimum acceptable diet intake among children age 6–23 months

**Fig. 4:**
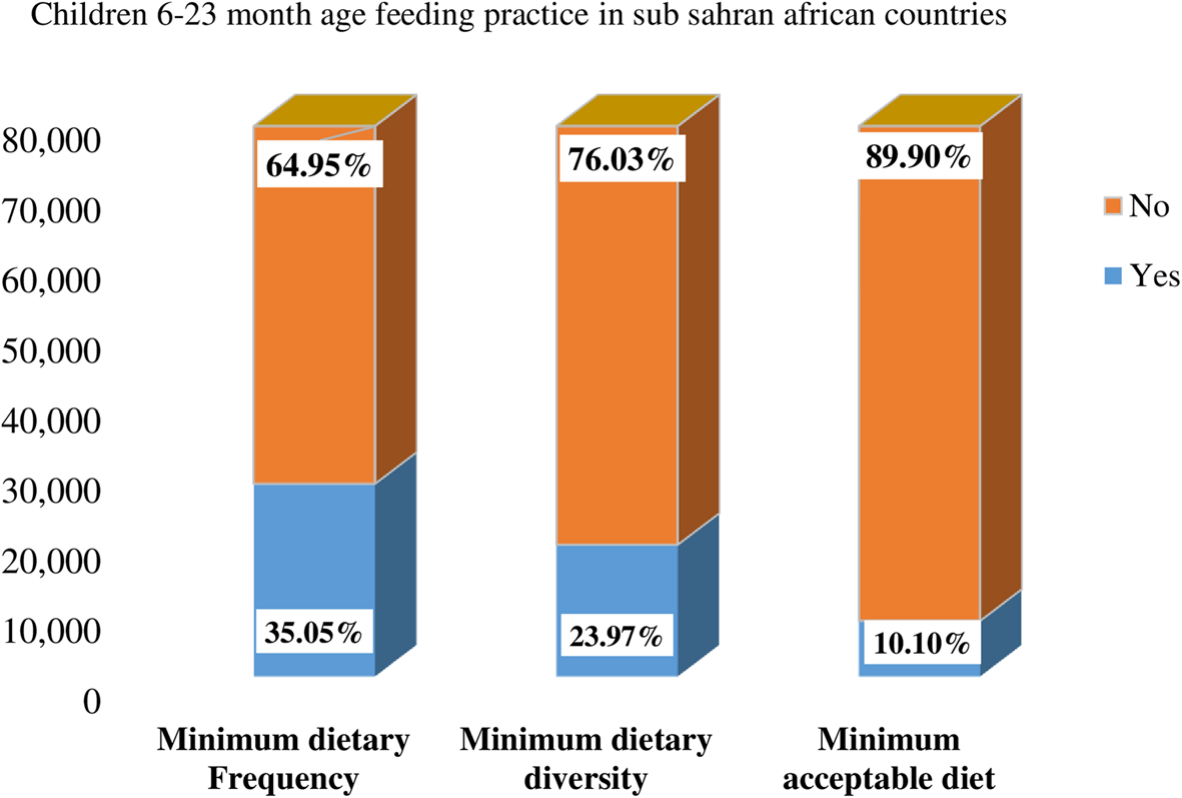
The bar graph shows the proportion of MMF, MDD and MAD intake among 6–23. Children across Sub-Sharan Africa countries using the recent DHS between 2010 to 2020

**Fig. 5:**
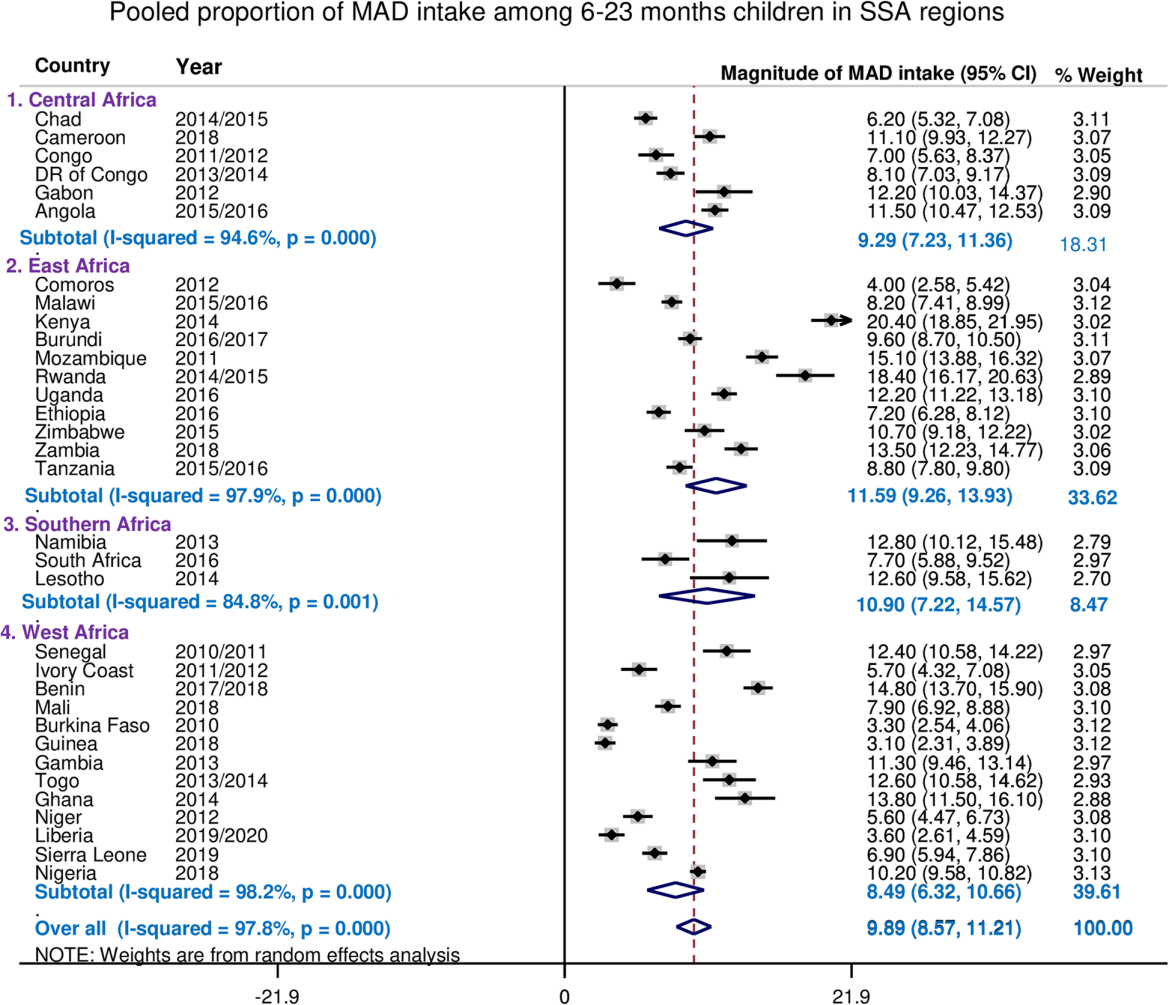
The Forest plot showed that pooled magnitude of MAD usage among 6–23 children in SSA

The overall pooled estimate of the minimum acceptable diet intake among children aged 6–23 months in Sub-Saharan African countries was 9.87% (95%CI: 8.57, 11.21%), with I^2^ = 97.8% and ranges from 3.10% in Guinea to 20.40% in Kenya (Fig. 5). Children aged 6–23 months in Sub-Saharan African have better access for meal frequency as compared to dietary diversity (Fig. 4).

Since the I^2^ value was large, which shows the true variabilities among 33 countries (the variability not by chance), then to treat the heterogeneity effect further subgroup analyses were performed based on the region in SSA, level of income of the country and the DHS survey year. The pooled magnitude of MAD intake ranges from 8.49% (95%CI: 6.32, 10.65%) in West Africa across 13 countries to 11.59% (95%CI: 9.26, 13.93%) in East Africa 11 countries based on subgroup analysis of regions in SSA (Fig. 5).

Moreover, the pooled magnitude of MAD intake across country income level was determined. The pooled estimate of MAD intake in low-income countries was 8.99% (95%CI: 7.39, 10.59%), lower-middle-income countries 11.75%(95%CI: 8.96, 14.53%), and 10.96% across upper middle-income countries (95%CI: 8.84, 13.04%) [S1]. The pooled estimate of MAD intake in the DHS survey conducted before and in 2015 was 10.65% (95%CI: 8.83, 12.92), whereas in the DHS survey after 2015 was 9.09% (95%CI: 7.45, 10.71) [S2].

Since the heterogeneity (I^2^) was not managed by doing subgroup analysis, further sensitivity analyses were performed to identify which country estimates were more contributed for the heterogeneity. Among 33 SSA countries using “in and out” of the single country which has deviant estimate at a time, seven countries (Kenya and Rwanda from the highest estimate and Guinea, Burkina Faso, Comoros, Liberia, and Chad from the lowest estimate) which had distant estimate from the pooled magnitude were dropped and cheeked the heterogeneity time by time. Then the remained 26 SSA countries pooled estimate was 10.29% (95%CI: 9.26, 11.31) with I^2^ = 94.8% (*p*-value = 0.008), which was still heterogeneous [S3].

### Multilevel model parameter results

#### Random effect and model comparison

As shown in Table 2, the ICC in the null model was 0.194, indicating that approximately 19% of the variations in minimum acceptable diet intake among children aged 6–23 months were attributed to cluster differences, while the remaining 81% were attributed to individual child factors.

The MOR value of 2.33, in the null model, also revealed that the odds of taking the minimum acceptable diet among study subjects were different between clusters.

Furthermore, the PCV value, 0.225 in the final model, indicates that about 22.5% of the variation in minimum acceptable diet intake among study subjects was explained by the final model (model four). Regarding model comparison/fitness, deviance was used. The model with the lowest deviance was the better fitted model, which was model four (45544) and selected for interpretation (Table 3).

**Table 3:** Parameters and model fit statistics for multilevel regression analysis models

Parameters	Null model	Model 2	Model 3	Model 4
Cluster level Variance	0.79	0.76	0.72	0.63
ICC	0.194	0.188	0.179	0.161
MOR	2.33[2.22, 2.41]**	2.29	2.24	2.12
PCV	Reff	0.038	0.089	0.225
Model fitness				
Deviance	50,378	45,762	49,528	45,544
Mean VIF	–	1.45	1.39	1.54

*ICC* Inter cluster correlation coefficient, *MOR* Median odds ratio, *PCV* proportional change in variance, *VIF* Variance Inflation Factors

^*^P-value < 0.05

^**^P value < 0.01

^***^P value < 0.001

#### Multi-level analysis of factors associated with MAD intake among 6–23 children in SSA

All variables (both individual level and community level variables) have a *p*-value< 0.20 in the bivariable analysis and eligible for multivariable analysis.

Based on the final model result, primary and above primary educated women were 1.09 and 1.41 times more likely to have MAD taken children than women with no formal education [AOR = 1.09; 95%CI; 1.01, 1.17] and [AOR = 1.41; 95%CI; 1.29, 1.53] respectively. The odds of having children with MAD were1.25 times more likely among women who have work than not working [AOR = 1.25; 95%CI; 1.17, 1.33]. Women who were not married were 21% times less likely to have children with MAD as compared to those who were married [AOR = 0.79;95%CI; 0.74, 0.84].

The odds of having children with MAD were 55% more likely among households which have exposure to at least one media (radio, TV or newspaper) than not exposed [AOR = 1.55; 95%CI; 1.45, 1.66]. When we goes from poorer to the richest households the usage of MAD among children increased by 13%, 26,37 and 93%as compared to the poorest households [AOR = 1.13; 95%CI; 1.04, 1.24], [AOR = 1.26; 95%CI; 1.15, 1.38], [AOR = 1.37; 95%CI; 1.25, 1.51] and [AOR = 1.93; 95%CI; 1.73, 2.15] for poorer, middle, rich and richest households respectively. Children from lower middle income and higher middle-income level countries also have 1.31 and 1.62 times more likely to access MAD than a child from lower-income level countries [AOR = 1.31; 95%CI; 1.25, 1.43] and [AOR = 1.62; 95%CI; 1.41, 1.87] respectively.

Children whose age found 12–23 months, were 1.62 times more likely to access MAD as compared to a child with 6–8 months of age [AOR = 1.62; 95%CI; 1.50, 1.75]. Being multiple births were 42% less likely to access MAD than those who were singleton [AOR = 0.68; 95%CI; 0.56, 0.82]. The odds of having MAD among children who are currently breast feed were two times higher than from those non-breast feed. [AOR = 2.04; 95%CI; 1.89, 2.21].

Women who had unwanted pregnancy for the last child were 10% times less likely to give MAD for their child as compared to those who had wanted pregnancy [AOR = 0.90; 95%CI; 0.85, 0.96]. The odds of having access to MAD of the child were 1.61 times higher among mothers who have at least one ANC follow-up [AOR = 1.61; 95%CI; 1.47, 1.77].

Children who lived in rural residence were 26% less likely to access MAD than those who lived in urban [AOR = 0.74; 95%CI; 0.69, 0.79]. Children who lived in the East Africa region had also 1.35 times more likely to access MAD as compared to those who lived in Central Africa [AOR = 1.35; 95%CI; 1.24, 1.47] (Table 4).

**Table 4:** Multilevel analysis of factors associated with MAD intake among children aged 6–23 months in Sub-Saharan Africa: based on 2010 to 2020 DHS

Variables	Categories	^a^Model 2	Model 3	Model 4
		AOR [95% CI]	AOR [95% CI]	AOR [95% CI]
Age of women (years)	15–19	1.00	**–**	1.00
	20–35	0.89 [0.79, 0.99]**	**–**	0.90 [0.78, 1.01]
	36–49	1.01 [0.88, 1.13]	**–**	0.92 [0.82, 1.05]
Sex of household head	Male	1.00	**–**	1.00
	Female	0.93 [0.87, 1.00]	**–**	0.93[0.87, 1.01]
Educational attainment of women	No education	1.00	**–**	1.00
	Primery education	1.23 [1.14, 1.32]***	**–**	1.09[1.01, 1.17]**
	Secondary&above	1.68 [1.56, 1.82]***	**–**	1.41 [1.30,1.54]***
Occupation of women	Not worked	1.00	**–**	1.00
	Worked	1.20 [1.13, 1.27]***	**–**	1.25 [1.17,1.33]***
Marital status of mother	Married	1.00	**–**	1.00
	Not married	0.82 [0.77, 0.88]***	**–**	0.79 [0.78,0.84]***
House hold family size	1–4	1.00	**–**	1.00
	5–10	1.06 [0.99,1.13]	**–**	1.07 [0.99, 1.15]
	> 11	1.12 [1.02,1.24]*	**–**	1.09 [0.97, 1.21]
Media exposure	No	1.00	**–**	1.00
	Yes	1.61 [1.51, 1.71]***	**–**	1.55 [1.45,1.66]***
Wealth index	Poorest	1.00	**–**	1.00
	Poorer	1.12 [1.02, 1.22]*	**–**	1.13 [1.04, 1.24]*
	Middle	1.26 [1.15, 1.37]***	**–**	1.26 [1.15,1.38]***
	Richer	1.43 [1.31, 1.57]***	**–**	1.37 [1.25,1.51]***
	Richest	2.11 [1.92, 2.31]***	**–**	1.93 [1.73,2.15]***
Birth order	<=3	1.00	**–**	1.00
	> 3	0.98 [0.91, 1.04]	**–**	0.98 [0.91, 1.05]
Sex of child	Male	1.00	**–**	1.00
	Female	1.05 [1.00, 1.11]	**–**	1.05 [0.99, 1.10]
Age of child	6–8	1.00	**–**	1.00
	9–11	1.00 [0.91, 1.10]	**–**	0.99 [0.90, 1.09]
	12–23	1.35 [1.26, 1.46]***	**–**	1.62 [1.50,1.75]***
Plurality of birth	Single	1.00	**–**	1.00
	Multiple	0.67 [0.55, 0.81]***	**–**	0.68 [0.56,0.82]***
Current breast feeding	No	1.00	**–**	**1.00**
	Yes	1.99 [1.85, 2.15]***	**–**	2.04 [1.89,2.21]***
Wanted pregnancy	Wanted	1.00	**–**	1.00
	Unwanted	0.92 [0.86, 0.97]**	**–**	0.90 [0.85,0.96]***
Place of delivery	Home	1.00	**–**	1.00
	Health institution	1.03[0.95, 1.09]	**–**	1.04 [0.98, 1.12]
ANC visit	No ANC visit	1.00	**–**	**1.00**
	At least one visit	1.65 [1.50, 1.81]***	**–**	1.61[1.47, 1.77]***
Community level variables				
Residence	Urban	**–**	1.00	1.00
	Rural	**–**	0.55[0.52,0.58]***	0.74 [0.69,0.79]***
Region in SSA	Central Africa	**–**	1.00	1.00
	East Africa	**–**	1.71 [1.58,1.85]***	1.35 [1.24,1.47]***
	West Africa	**–**	1.17 [0.07,1.27]***	1.05 [0.96, 1.15]
	South Africa	**–**	1.05 [0.88, 1.24]	0.93 [0.76, 1.12]
Country income level	Lower	**–**	1.00	1.00
	Lower middle	**–**	1.31 [1.24,1.40]***	1.33[1.25,1.43]***
	Upper middle	**–**	1.28 [1.12,1.45]***	1.62[1.41,1.87]***
Survey year	2010–2012	**–**	1.00	1.00
	2013–2015		1.24[1.14,1.34]***	0.98 [0.90, 1.07]
	2016–2019		1.09 [1.02, 1.19]**	0.94 [0.86, 1.02]
Community media usage	Low	**–**	1.00	1.00
	High	**–**	1.19[1.12,1.27]***	0.99 [0.93, 1.07]
Community women education	Low	**–**	1.00	1.00
	high	**–**	1.07 [1.00, 1.14]*	0.99 [0.92, 1.06]

*AOR* Adjusted Odds Ratio, *CI* Confidence Interval

^a^Model 1(null model) = the model which contain only with dependent variable and values expressed in Table 4

^*^P-value < 0.05

^**^P value < 0.01

^***^P value < 0.001

## Discussions

Inadequate infant and young child feeding (IYCF) practices are the most important global problems and the major determinants of under nutrition, optimal growth, and development, especially in the first 2 years of life [4]. Identifying and reducing avoidable determinants of malnutrition is a critical step toward improving children’s overall health and well-being [40]. This study aimed to determine the pooled estimates of minimum acceptable diet intake and to identify associated factors among children aged 6–23 months in Sub-Saharan Africa. Based on this, only 9.89% of children aged 6–23 months in Sub-Saharan African countries can access a minimum acceptable diet in this study. It ranges from Guinea (3.10%) to Kenya (20.40%). This is in line with a research conducted in North-Eastern, Eastern, and Southern regions of India which was 9% [15]. But lower than a multi-site study conducted in America, Asia, and Africa 21% [41], South Asia countries [21], Bangladesh 20% [17] and Indonesia 40% [16] of children aged 6–23 can access minimum acceptable diet. The discrepancy might be due to geographical variation, population growth, and socio-economic status of the countries [41]. Cultural beliefs and knowledge paradigms about MAD are also known to influence feeding practices [5, 15]. Studies showed that growth faltering among Sub-Saharan African children becomes evident from early infancy and sustained through the second year of life which is the period with the highest reported prevalence of overall malnutrition [42]. Governmental actions toward the application of national nutritional programs, and addressing cultural beliefs around complementary feeding is also one of the crucial difference [22]. For instance, the better magnitude of MAD intake in Kenya was achieved by implementing a health platform which is called the Baby Friendly Community Initiative platform and by integrating WASH (Water, Sanitation and Hygiene) into complementary feeding sessions [22]. The availability and accessibility of foods in the region may have also a contribution. Children in agrarian dominant and city dwellers were more likely to have MAD [13, 25, 29]. For instance. Guinea is among the poorest countries in the world which ranks 179 of 187 countries with 10% population were food insecure [43]. But our finding is higher than a study conducted and in the Philippines 6.7% [14] of children age 6–23 can access a minimum acceptable diet. This is due to that, current study included a large population from different geographic Sub-saharan African regions with various cultures, beliefs, and traditions which make it a real estimation of the magnitude in SSA.

The results of this study also demonstrate that an array of sociodemographic and health factors are associated with a minimum acceptable diet feeding in Sub-saharan Africa. The current study found that children 12–23 months of age had higher odds of meeting the minimum acceptable diet, implying that the practice of MAD significantly increases as the child’s age increases. This is in line with studies conducted in Bangladesh [22], Indonesia [13], Malawi [12] and different districts of Ethiopia [20, 25]. Children in the youngest age (6–11 months) in Benin and Burkina Faso, Guinea, Niger and Senegal also had a significantly high risk of not meeting the requirement for MAD [23]. This may be due to due to the late introduction of complementary feeding and when they start complimentary feeding on time; they included only milk or cereal products [23, 25]. Another possibility could be mothers may perceive that, younger the children, the poor ability of the intestine to digest certain foods like banana, egg, pumpkin, carrot, green vegetables and meat [44]. Loss of appetite during teething and weight loss due to increased infections could explain changes in feeding practices among children 6 to 8 months aged [45].

According to this study, as women’s educational status rises, so do their chances of having children who use MAD. This is in line with a study done in South Asia [21], India [15], Indonesia [13], Bangladesh [22], Ghana [46], Tanzania [24] and Ethiopia [20, 26, 28, 47]. However, studies from Filipino [14] and Ethiopia [10] reported that there was no significant association between higher maternal education and complementary feeding practices. This difference could be due to differences in the sample size. Mothers’ knowledge of child feeding was positively associated with minimum dietary diversity in child feeding practice [5, 19, 26]. Owing to the fact that educated women are more likely to have access to quality health services and messages, which they may more easily comprehend and translate that information into practice [12, 44]. Furthermore, promotion of optimal child nutrition, and subsequent uptake of health information and health-seeking behavior of educated mothers [15].

In this study, women who are currently not married were less likely to have children with access to MAD as compared to those who were married. This is in line with research conducted in India [15], Indonesia [13], and Ethiopia [48]. This might due to that single mothers have lack support from families or communities, which causes poor infant feeding practices [13, 49].

In our study, children aged 6–23 months children whose mothers were working have more likely to get MAD. This is supported by a study conducted in Indonesia [13], West African countries [23], Malawi [12], Tanzania [24], Ethiopia [10, 25], but other studies showed that mother occupation have not significant association with getting a child MAD [5, 19]. The later studies may have differed because they were conducted in a single district with small sample size. The possible explanation for our finding is that women with formal employment may be in a better socioeconomic position to achieve food security, which in turn may influence their complementary feeding practices [12]. Employed mother mostly has a higher education status which eventually leads the household decision-making process to purchase and feed the type of food that is necessary for their child [24]. A mother’s earning ability, access to resources, wider social networks and a growing understanding of their social environment could increases opportunities to feed the child minimum acceptable diet in working mothers [24, 25] On the other hand when mothers do not work outside the home, they have more time to breastfeed the child without thinking of giving them complementary food [23].

In this study, we found that children whose mothers have access to the media had a significantly higher chance of meeting the minimum acceptable diet requirement This is in line with a study in South Asia [21], India [15], West African countries [23], Malawi [12] and Ethiopia [5, 10, 20, 25, 26, 28]. This could be attributed to the media, which is generally regarded as a reliable source of health and nutrition information, and thus its messages are more likely to be adopted [12]. As a result, mothers who are exposed to the media may gain knowledge and effect behavioral change by providing their children with a minimum acceptable diet.

In our study, the odds of fulfilling MAD criteria among children who are currently breast feed are two times higher than those non-breast feed. This is in line with EDHS 2016 report which shows the proportion fed according to the minimum acceptable dietary standards is somewhat lower among non-breastfed children than among breastfed children [29]. This is because of that, in our study only 41% of no breastfed children are fed with milk or milk products as recommended. In addition, the recommended meal frequency is lower for breastfeed children so as can easily access the expected frequency of MAD [2, 6, 9, 11].

People who had multiple births were less likely to feed MAD to their children than singletons in this study. It is supported by a study in Nigeria which showed, children of multiple births are more likely to be stunted than those of single births [27]. Another study in Japan also showed that mothers with twins or triplets were twice as likely to choose bottle-feeding with formula milk only than mothers with singletons [50]. This could be due to that women with single children may commit themselves to taking better care of their children and feeding them more appropriately with respect to complementary feeding practice [12]. Inadequate breastfeeding and competition for nutritional intake, on the other hand, occur more frequently in children of multiple births than in children of single births [27].

In our study, children from mothers who wanted to be pregnant had a higher chance of having access to MAD than those who were unwanted. This is consistent with previous Bangladesh research [51]. A study in central New York State showed that women with mistimed pregnancies and pregnancies that were not wanted were significantly less likely to breast-feed than women who had planned pregnancies [52]. A study, on the other hand, found that optimal childcare and breastfeeding are better in the desired pregnancy than in an unwanted pregnancy [53]. The reason might be, mother’s attitudes and behavior; her feelings about having an unwanted child might contribute to conscious or unconscious neglect of the child, reduce her ability to cope with a young child’s everyday needs, and diminish the quality of her caregiving behaviors, leading to malnutrition for the child [51]. Women with unwanted pregnancies can be victims of severe mental illness and postpartum depression due to their unsuitable feelings and emotions, which can lead to a decrease in their maternal role especially in breastfeeding [54].

According to this study, if mothers have antenatal care visits (ANC) during their pregnancy, their child has a higher chance of having MAD. This is in line with a study conducted in South Asia [21], India [15], seven francophone West African countries [23], but not in other studies in Tanzania [24], Ethiopia [10, 20]. The disparity could be explained by the fact that the studies that show a significant association with MAD are numerous sample sizes, or by the fact that the latter is a single country with single district studies. But generally, our findings may be attributed to several factors. First, effective nutrition education and counseling (often provided during ANC visits) might contribute to MAD usage. Second, low maternal education, the cost associated with attending ANC service, and a lack of awareness of the importance of ANC are factors related to non-use of ANC service which eventually leads to low awareness and usage of MAD intake [15].

Children who lived in rural areas were less likely to meet the minimum acceptable dietary standards in this study. This is in line with a study conducted in Indonesia [13], South Asia [21], seven francophone West African countries [23], and a report of EDHS 2016 [29]. However, studies in the Philippines and District Ethiopia found no link between residence and MAD usage [5, 14]. The discrepancy might be due to the small sample size of the later studies. The reason for having a higher chance of using MAD in the urban residence might be the result of the cumulative effect of a series of more favorable conditions, including better socioeconomic and educational conditions, in turn leading to better caring practices for children and their mothers [55, 56].

In this study, we found that as the wealth status of the household’s increases from poorer to richest, the chance of having children who use MAD significantly increases. Children from lower-middle and higher middle-income level countries also have more access to MAD as compared to lower-income levels. This is in line with a study conducted in, India [15, 57, 58], Bangladesh [22], South Asia [21], Tanzania [24], and Ethiopia [19, 28, 59], but other studies in North Shoa, and Mareka District, Ethiopia showed that no association with it [5, 20]. This difference might be due to the small sample size and covers the small district of the latter two studies. But it is known that children’s from a family of higher income can feed diversified foods and frequently as their families could be more likely to afford to have diversified foods as compared to children from a low household income [55].

The main strength of this study was the use of the weighted nationally representative data of each Sub-saharan African country with a large sample which makes it representative at Sub-Saharan and regional levels. Therefore, it has appropriate statistical power that can be generalized of the estimates in minimum acceptable diet intake in the study setting to all children 6–23 months aged during the study period. Another strength of this study was estimating the pooled estimate of minimum acceptable diet intake in Sub-Sharan Africa and sub-regions will give invaluable information for region-specific intervention. Since the data were collected cross-sectional at a different point in time by self-reported interview would be prone to recall and social desirability bias. The drawback of the secondary nature of data was inevitable. The heterogeneity of the pooled estimate of MAD intake was not managed by further analysis.

## Conclusion

The proportion of minimum acceptable diet usage among children aged between 6 and 23 months in Sub-saharan Africa was relatively low. Individual level factors such as, having an educated mother, an employed mother, having media exposure, increasing the wealth status of the household, being 12–23 months aged child, currently breast feeding child, have a positive association with MAD usage of the child. Whereas having an unmarried mother, unwanted pregnancy, and having multiple births have a negative association with MAD usage among children aged 6–23 months. From community level variables living in lower and upper middle income level of the country, and living in East Africa region had positive association whereas, living in rural residences have a negative association with MAD usage among children aged 6–23 months in Sub-Saharan Africa.

## Recommendation

To increase minimum acceptable diet intake among children aged 6–23 months in Sub-Saharan Africa, policymakers in nutritional projects and other stakeholders should work as an integrated approach with other sectors, and give prior attention to modifiable socio-economic factors such as promoting women’s education and employment, increase wealth status and media exposure of the household, and promoting breast feeding behavior.

### For sub-Saharan Africa countries Ministry of Health

It is recommended to promote breast feeding of children aged 6–23 months as per the WHO recommendation. A program to establish feeding practices for multiple births should be developed. Child nutrition and feeding guidance for mothers need to be especially targeted to lower-educated mothers. Promoting feeding of children with MAD through social and behavior change interventions which targeting children aged 6–8 months, multiple births, and rural residences and children from unmarried mothers. Child nutrition program planners should consider utilizing mass media for delivering important messages.

### For sub-Saharan Africa countries Ministry of Education

The ministers of countries should empower and increase the number of educated mothers. Mass media is essential in delivering important messages, thereby imparting necessary knowledge to mothers. Therefore enhance the accessibility of media and use to delivery health education.

### For the government of sub-Saharan Africa countries

Should plan and work to enhance their country’s economy to the middle and higher economic level and to improve the wealth index of individual households.

## Supplementary Information

**Additional file 1: S 1.** Sub group analysis of pooled proportion of MAD usage based on country income status.

**Additional file 2: S 2.** Sub group analysis of pooled proportion of MAD usage based on DHS survey year.

**Additional file 3: S 3.** Sensitivity analysis after omitted seven counties which have deviant estimate.

## Data Availability

Data is available publically access from the open databases. It can be accessed by the following website https://dhsprogram.com/data/dataset_admin/login_main.cfm?CFID=10818526&CFTOKEN=c131014a480fe56-4E0C6B7F-F551-E6B2-50
