# Supplementary figures and images for: Minimum acceptable diet intake and its associated factors among children age at 6–23 months in sub-Saharan Africa: a multilevel analysis of the sub-Saharan Africa demographic and health survey
Source: BMC Public Health. 2022 Apr 7;22:684. doi: 10.1186/s12889-022-12966-8 (PMC8991979; doi:10.1186/s12889-022-12966-8)

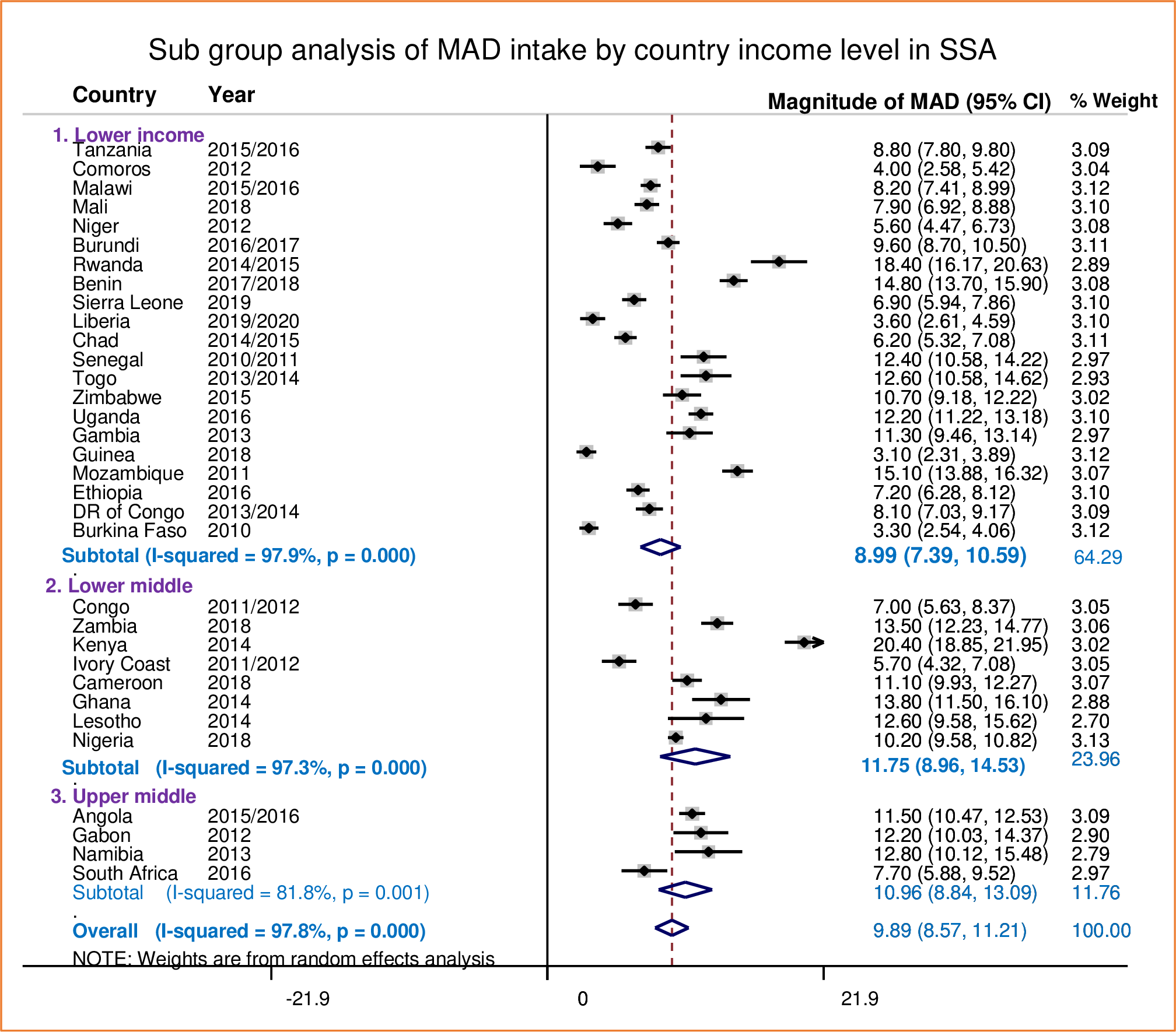

Supplement: Supplementary file 1 — Additional file 1: S 1. Sub group analysis of pooled proportion of MAD usage based on country income status. [file 12889_2022_12966_MOESM1_ESM.tif]

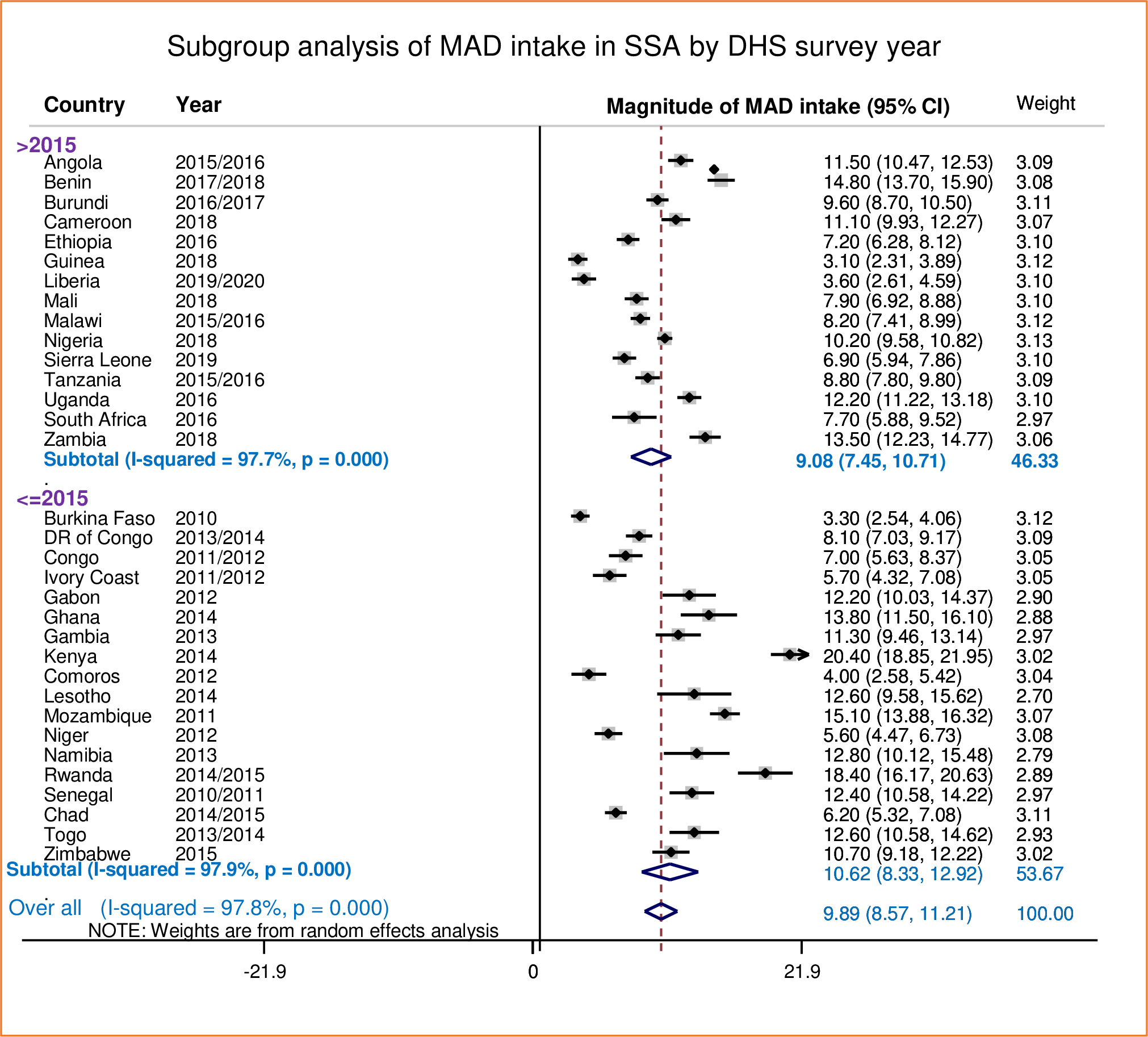

Supplement: Supplementary file 2 — Additional file 2: S 2. Sub group analysis of pooled proportion of MAD usage based on DHS survey year. [file 12889_2022_12966_MOESM2_ESM.tif]

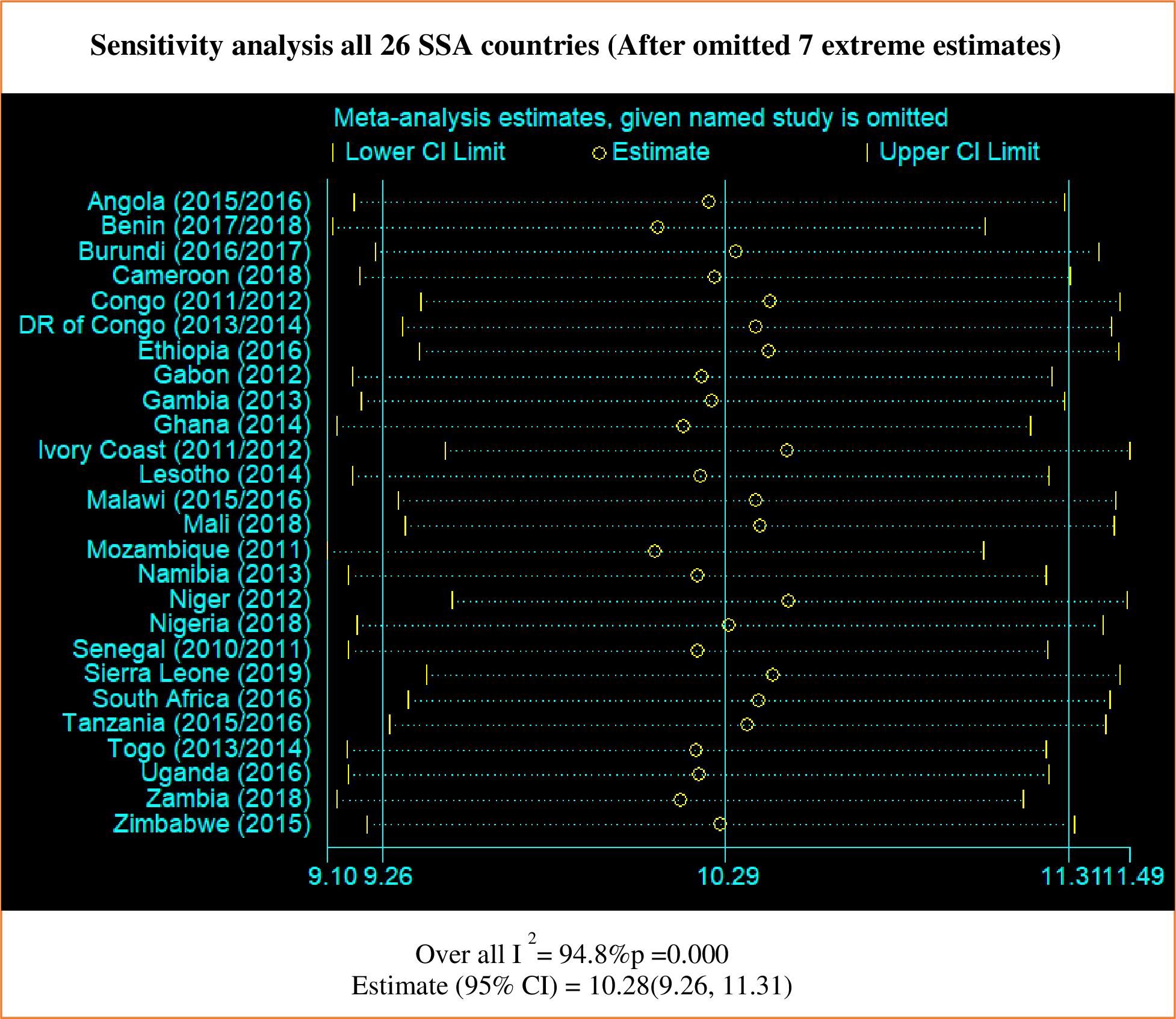

Supplement: Supplementary file 3 — Additional file 3: S 3. Sensitivity analysis after omitted seven counties which have deviant estimate. [file 12889_2022_12966_MOESM3_ESM.tif]
